# Investigative Method for Fatigue Crack Propagation Based on a Small Time Scale

**DOI:** 10.3390/ma11050774

**Published:** 2018-05-11

**Authors:** Hongxun Wang, Weifang Zhang, Jingyu Zhang, Wei Dai, Yan Zhao

**Affiliations:** School of Reliability and Systems Engineering, Beihang University, Haidian District, Beijing 100191, China; wanghongxun@buaa.edu.cn (H.W.); zhangweifang@buaa.edu.cn (W.Z.); jingyuzhang@buaa.edu.cn (J.Z.); zy_buaa@buaa.edu.cn (Y.Z.)

**Keywords:** fatigue crack propagation, small time scale, in-situ SEM, 7050-T7451, microstructure

## Abstract

In-situ scanning electron microscopy (SEM) testing based on a small time scale is proposed to integrally investigate the fatigue crack growth behavior and mechanisms, which is different from the widely-used, cycle-based approach due to its small time scale and comprehensive analysis of the effects of microstructure, crack closure and applied loading on crack growth. In the proposed methodology, the behavior of fatigue crack growth at any time within a loading cycle is observed by SEM to investigate the influence of microstructure on crack growth. Images with high resolution are taken to measure the crack tip opening displacement (CTOD), and the correlation between CTOD and the stress intensity factor (SIF) *K* is studied. A model based on experimental data is used to predict the CTOD variation. The unstable crack growth of aluminum alloy 7050-T7451 is investigated using the proposed method. Results show that this method has great potential in fatigue crack growth mechanism research compared with the traditional cycle-based approach.

## 1. Introduction

The fatigue damage accumulation process is a multi-scale phenomenon, which includes different spatial and temporal scales [1]. The fatigue life includes two phases during cyclic loading: crack initiation followed by a crack growth period until failure. For the temporal scale, fatigue analysis methods have mainly focused on the computation of damage based on several loading cycles, and the time scale of this cycle-based approach is mainly from ten loading cycles, hundreds of loading cycles or even thousands of loading cycles [2,3,4,5]. The time and spatial scales of the cycle-based approach are relatively large, which limits the recognition of fatigue damage mechanisms. Additionally, the cycle-based approach has many other difficulties in fatigue analysis. Firstly, the material stress state cannot be expressed in detail through the mechanical driving force of the cyclic range using the cycle-based approach, which causes the stress ratio effect when specifying material fatigue properties. Secondly, it is also difficult to analyze the damage mechanism owing to the inconsistency of the time scale when the fatigue damage is connected to other time-based types of damage. Furthermore, the time history of loading is transformed to the cycle history in the cycle-based approach of traditional fatigue analysis, which has many additional uncertainties because not all information is transformed during cycle counting [6]. Previous investigations have shown that the time–based approach is feasible to analyze the fatigue damage mechanism. Lu and Liu [1] proposed a new small time scale method for analyzing the behavior of fatigue crack propagation. The small time scale method was based on the incremental crack growth at any time instant (*da*/*dt*) within a loading cycle instead of the reversal-based crack growth rate (*da*/*dN*) [7]. There are two hypotheses regarding the small time scale method; one is that cracks only grow during the loading path, another is that no cracks grow during the unloading path [6].

The implementation of the small time scale method needs to be based on in-situ scanning electron microscopy (SEM) testing, which is widely used to investigate the fatigue crack initiation and propagation behaviors of alloys, such as IN718 alloy [8] and DZ4 superalloy [9]. Chai [10] investigated the effect of crack branches on the crack growth rate of duplex stainless steel SAF 2507 within several cycles using the in-situ SEM method. Qiu [11] observed the behavior of crack closure and measured the crack tip opening displacement (CTOD) *δ* of the nickel-based SC superalloy during the unloading process using the in-situ SEM method. Zhang [12] studied the relationship between the CTOD and the growth rate of short and long crack of aluminum alloys by in-situ investigation of small fatigue crack growth. Elber confirmed the behaviors of crack opening and closure, but the quantitative analysis method of crack opening and closure was not proposed. Elber’s model and its modified models are used to calculate the crack opening stress level, but these models usually introduce hypothetic intermediate parameters, which do not have physical meaning [13].

High strength aluminum alloys are widely used in the aerospace field due to their good integrated performance, and it is crucial to study the fatigue damage for these materials during work in severe environments when considering the safety of structures. It has been indicated that the fatigue crack growth of aluminum alloys is closely related to many factors, such as the applied loading, microstructure and crack closure. However, there is no appropriate method to comprehensively investigate these factors at the same time. The in-situ SEM testing based on a small time scale can study the microstructure, CTOD, crack opening and closure, which provides the feasibility to comprehensively investigate crack growth behavior and mechanisms. Moreover, the crack opening stress level and CTOD can be measured directly by in-situ SEM testing, and the parameters all have physical meaning. In this study, in-situ SEM testing based on a small time scale is used to investigate the correlations between applied loading, CTOD and crack opening and closure, and to analyze the effect of microstructure on the fatigue crack growth in aluminum alloy 7050-T7451 under constant amplitude loading.

In this study, the method of in-situ SEM testing based on a small time scale is proposed, and the crack growth behavior and mechanisms of aluminum alloy 7050-T7451 are integrally analyzed by this method. The paper is organized as follows. Firstly, the methodology of the in-situ SEM testing based on a small time scale is introduced. Secondly, the specimen and experimental procedure is provided. After that, a comprehensive investigation of the crack growth behavior and mechanism of aluminum alloy 7050-T7451 is completed. Finally, some conclusions and future works are summarized.

## 2. Methodology

### 2.1. In-Situ SEM Testing Based on Small Time Scale

**Figure 1:**
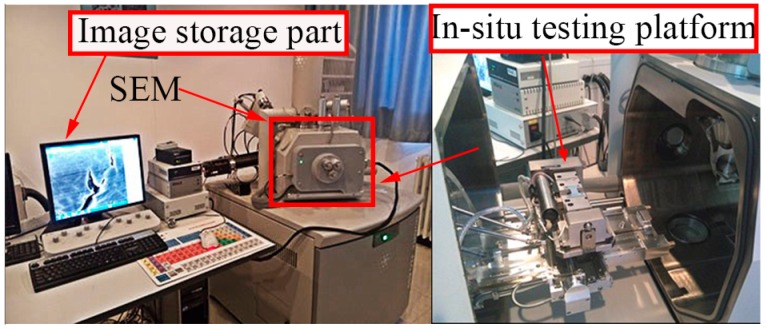
In-situ SEM fatigue testing system.

The experimental instrument for the in-situ SEM testing based on a small time scale includes a loading part and an observation and image storage part, as shown in Figure 1. The in-situ testing platform (Deben 2000) is driven by the servo motor, and the chucks of specimen are driven through the ball screw to load the specimen. The load capacity of the in-situ testing platform is 2 KN, and the maximum gage length between mechanical grips is about 27 mm. The testing platform is only suitable for the tensile test and low cycle fatigue test owing to the limited loading speed of the servo motor.

The testing platform is installed in the vacuum chamber of the SEM (Quanta 650, FEI, Hillsboro, OR, USA), and the vacuum degree of working chamber is lower than 6 × 10^−4^ Pa. During the process of the in-situ SEM testing, the loading can be stopped at any time, and the morphologies can be observed through the display screen, including scratches, inclusions and cracks and other surface features.

**Figure 2:**
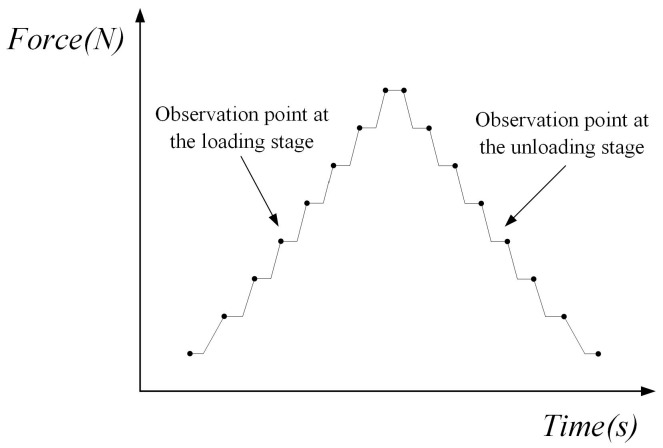
Schematic of small time scale loading process.

In-situ fatigue testing is generally carried out at room temperature, but it also can be equipped with heating devices for high temperature testing, and the maximum heating temperature is 800 °C. In the in-situ SEM experiment based on a small time scale, the single edge notched specimen is employed, and one loading cycle is decomposed with several loading and unloading steps, as shown in Figure 2.

### 2.2. Measurement for Crack Growth Opening Displacement

**Figure 3:**
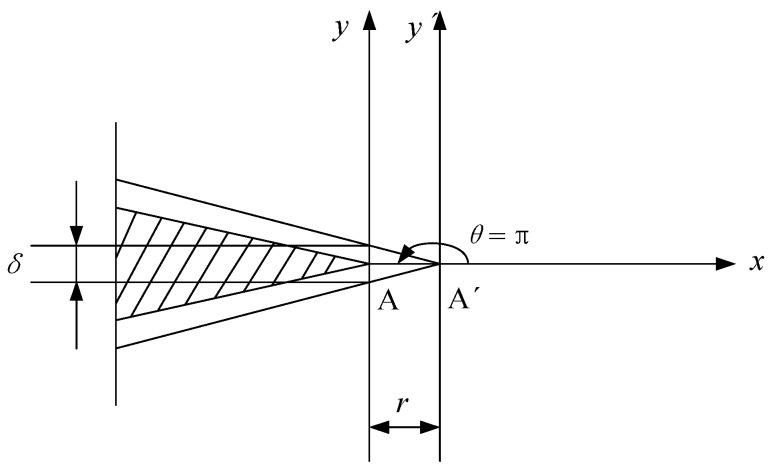
The first measurement method of the crack tip opening displacement (CTOD).

**Figure 4:**
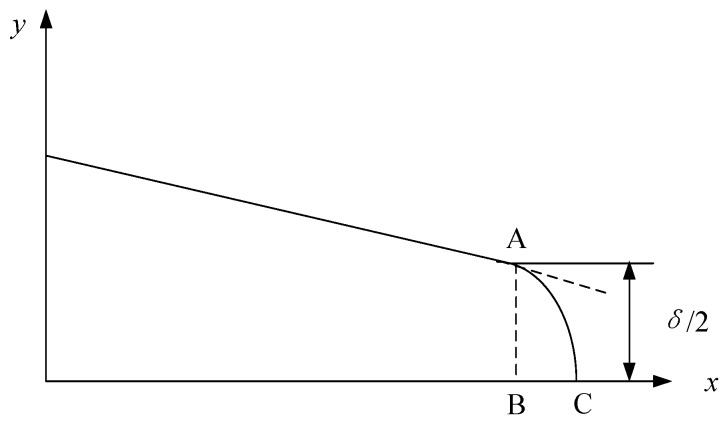
The second measurement method of CTOD.

**Figure 5:**
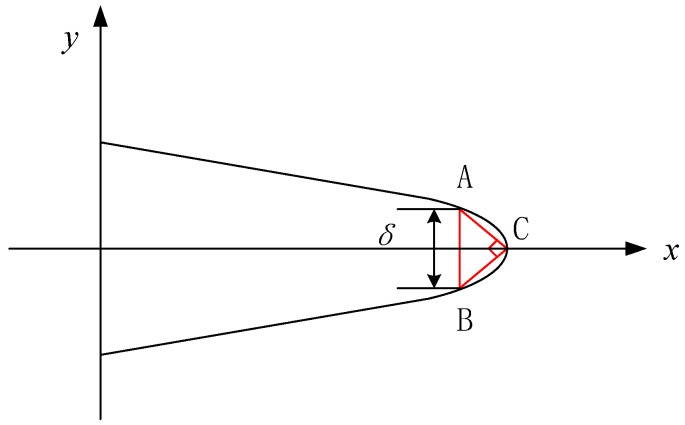
The third measurement method of CTOD.

In the small time scale method, CTOD *δ* is directly correlated with crack propagation. The stress intensity factor (SIF) *K* can characterize the material stress state near the crack tip. Thus, CTOD and SIF are used as measurement indexes to quantitatively describe the behavior of crack opening and closure. The main measurement methods of CTOD are defined as follows [14]:

1. In the plastic zone correction of linear elastic fracture mechanics, when the crack propagates, the origin crack tip of point A grows to point A’. The CTOD is defined as the displacement of the origin crack tip on the Y axis, as shown in Figure 3. However, it is not easy to determine the position of point A.
2. The second measurement method of CTOD is shown as Figure 4. The intersection point of the elastic zone and plastic zone on the crack surface is regard as point A. The displacement of intersection point A is the length of line AB, and the CTOD is double the length of line AB. However, the displacement is hard to measure effectively from the experiment.
3. Point C of the crack tip after growth is regard as the apex. Based on the original crack (X axis), the right triangle is formed, which is symmetrical to the origin crack. The opposite sides of the right triangle and the crack surface intersect two points, A and B, and the CTOD is the length of line AB, as shown in Figure 5. This measurement method of CTOD is currently widely used.

**Figure 6:**
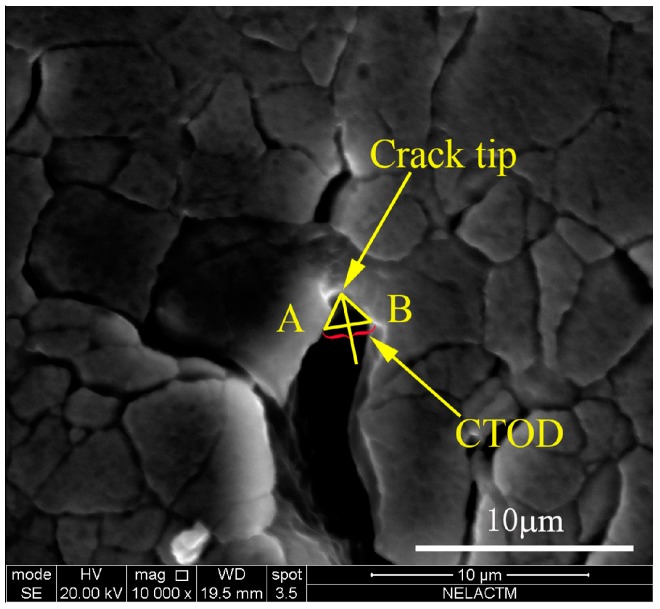
An example of the third method in the in-situ SEM testing.

Compared with the third measurement method of CTOD, the first method has difficulty finding the position of the origin crack tip. In the second method, it is difficult to measure the displacement of intersection point A of the elastic zone and plastic zone on the crack surface. However, the third method can more conveniently and accurately measure the CTOD. Therefore, the third method is used to measure the CTOD in this paper. Figure 6 is an example of the third measurement method of CTOD in the in-situ SEM testing.

### 2.3. Stress Intensity Factor

In fracture mechanics, SIF is generally used to characterize the stress intensity near the crack tip, which depends on the geometry of the specimen, applied loading and crack length. For the single edge notched specimen, the SIF at each loading step in a loading cycle under the plane stress state can be calculated from the empirical Equation (1) [13] and Equation (2) [15].

(1) $$K=F\sigma \sqrt{\pi a}$$

(2) $$F=\sqrt{\frac{2b}{\pi a}tan\frac{\pi a}{2b}}\frac{0.752+2.02(\frac{a}{b})+0.37(1-\sin\frac{\pi a}{2b}{)}^{3}}{\cos\frac{\pi a}{2b}}$$

where *F* is the geometry factor, *σ* refers to the stress, *a* is the crack length, and *b* is the width of the plate in the direction of the crack. It was reported in reference [15] that for any *a*/*b*, the accuracy of the Equation (2) is better than 0.5%.

### 2.4. Modeling of Crack Tip Opening Displacement

To investigate the correlation between CTOD variation and SIF in a loading cycle, the preliminary CTOD prediction model was established to describe experimental observations. In this study, the crack problem is for a through crack, which mainly occurs under plane stress; thus, the CTOD can be expressed by Equation (3) [1]:

(3) $$\delta =\frac{K^{2}}{2E\sigma_{y}},$$

where *δ* is the CTOD, *E* represents the Young’s modulus, and *σ_y_* represents the yield strength. The CTOD variation is consistent with the effective SIF according to Equation (3), and the crack closure also has an effect on the CTOD variation owing to the experimental investigation. Therefore, the CTOD variation modified by the crack closure level can be expressed by Equation (4) based on Equation (3) [6]:

(4) $$\delta =\left\{ \begin{array}{ll} \frac{1}{2\alpha }\frac{{\left(K-K_{op}\right)}^{2}}{E\sigma_{y}} & K\geq K_{op}\\ 0 & K<K_{op} \end{array}\right.,$$

where *α* is the material hardening parameter, *K* is the current applied SIF value and *K_op_* is the crack opening level.

## 3. Experimental Investigation for Aluminum Alloy 7050-T7451

### 3.1. Material and Specimen

In this study, the high strength aluminum alloy 7050-T7451 was used in the in-situ SEM testing. The aluminum alloy 7050-T7451 is widely used in aircraft structures due to its excellent properties, such as its high structural strength, fracture toughness and good stress corrosion resistance [16,17]. Its chemical composition and mechanical properties are measured and listed in Table 1 and Table 2, respectively.

**Figure 7:**
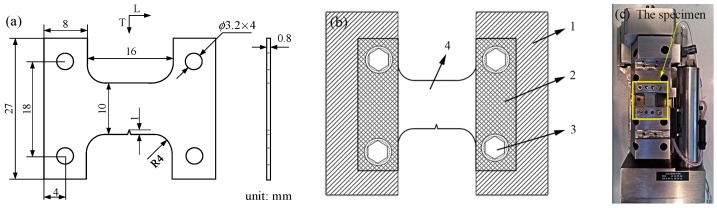
Specimen and installation: (**a**) the geometry of the specimen; (**b**) The installation of specimen: (1) the in-situ testing platform; (2) the press plate; (3) the inner hexangular set bolt; (4) he specimen; (**c**) The installation of specimen on the in-situ testing platform.

In this study, due to the method of installation of the specimen, the shape of the single edge notched specimen was designed based on the in-situ testing platform. The specimen with a width (W) of 27 mm, length (L) of 32 mm and thickness (B) of 0.8 mm is shown in Figure 7a. The edge notch of the length is 1 mm, and the angle of the edge notch is 30 degrees. The direction of the specimen’s length is consistent with the rolling direction of the material. As shown in Figure 7b, the specimen needs to be installed on the in-situ testing platform before the in-situ SEM testing. The specimen is compressed by the press plates; then the specimen and the press plates are both installed on the in-situ testing platform through the inner hexangular set bolts. Finally, the installation of specimen on the in-situ testing platform is shown in Figure 7c.

### 3.2. Experimental Setup

**Figure 8:**
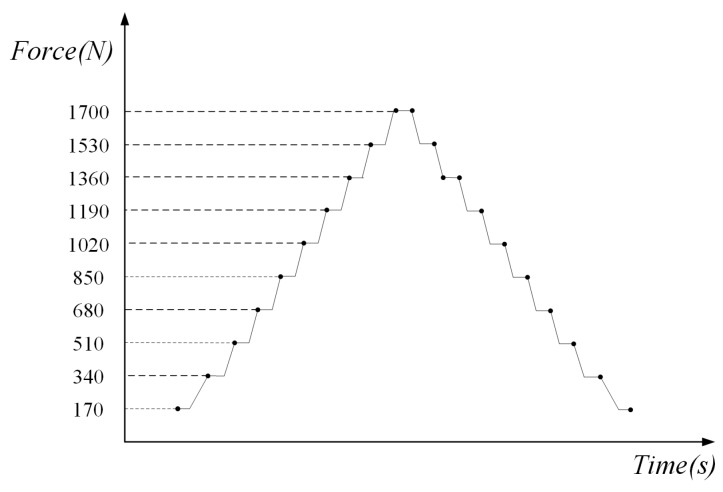
Schematic of the loading and unloading steps.

First of all, the specimens were pre-cracked on the fatigue testing machine (INSTRON-8801) until the initial crack length was 1 mm so that the fatigue crack could stably propagate in the following in-situ SEM testing. The experimental process followed ASTM standard E647-00. The stress ratio *R* of the pre-cracking was 0.1. After that, the surfaces of specimens were ground using abrasive paper with mesh numbers from 1000 to 2000 and polished utilizing a diamond polishing agent with a particle size of 2.5 µm. Then, the specimens were etched using Graff Sargent solution (1 mL HF + 16 mL HNO_3_ + 3 g CrO_3_ + 83 mL H_2_O) for 20 s. Following this, the specimen was installed and loaded on the in-situ testing platform and observed by using SEM. The maximum loading (*F*_max_) was 1700 N and the stress ratio (*R*) of the constant amplitude loading was 0.1. The loading speed was 0.1 mm/min. In this paper, a loading cycle was divided into 20 loading and unloading steps, as shown in Figure 8. The in-situ SEM testing was controlled by the loading speed which was 0.1 mm/min. The in-situ testing platform had the force sensor which could display the applied loading in real time. During the process of in-situ SEM testing, the platform would stop loading the specimen when the loading reached the set value. Then, the images of the crack tip under the magnification 10,000 were taken and saved to measure the CTOD within one loading cycle.

## 4. Results and Discussion

### 4.1. Fatigue Crack Opening and Closure

**Figure 9:**
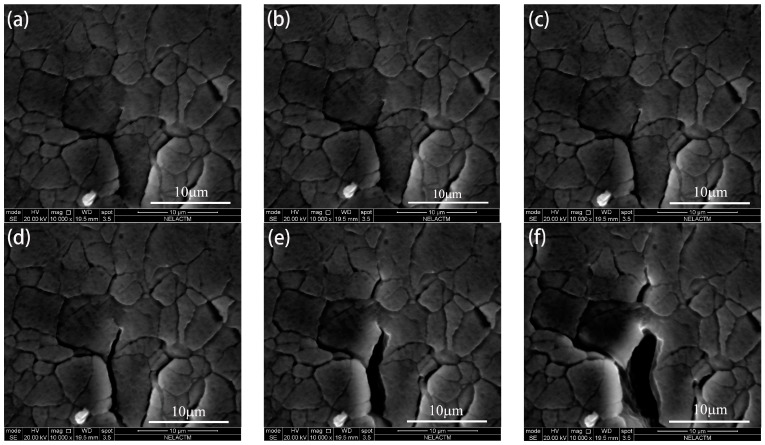
The behavior of fatigue crack opening during the loading steps: (**a**) *K* = 2.17; (**b**) *K* = 4.33; (**c**) *K* = 8.65; (**d**) *K* = 10.82; (**e**) *K* = 17.31; (**f**) *K* = 21.63 (units: MPa·m^0.5^).

The following images of fatigue crack opening and closure were selected from the experimental results to study the crack growth behavior. The behavior of the fatigue crack opening during the loading steps is shown in Figure 9. From Figure 9a,b, it can be observed that the fatigue crack remained closed at the initial loading step. As shown in Figure 9c, the crack started to open when *K* was 8.65 MPa·m^0.5^. From Figure 9d–f, it can be seen that CTOD increased with an increase in loading; thus, the maximum CTOD was achieved when the loading reached the highest level. Moreover, crack tip blunting is also observed, as shown in Figure 9f. It also can be seen that the crack propagation was accompanied by an increase in CTOD.

**Figure 10:**
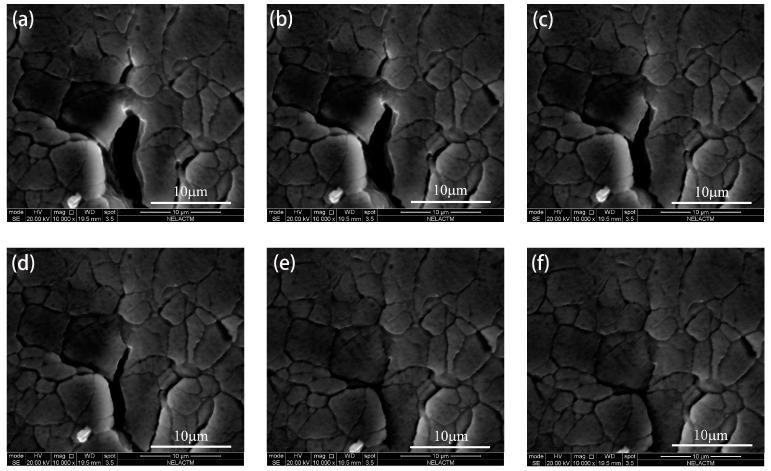
The behavior of fatigue crack closure during the unloading steps: (**a**) *K* = 21.63; (**b**) *K* = 17.31; (**c**) *K* = 10.82; (**d**) *K* = 8.65; (**e**) *K* = 4.33; (**f**) *K* = 2.17 (units: MPa·m^0.5^).

The behavior of fatigue crack closure during the unloading steps is shown in Figure 10. It can be seen that the crack closed gradually from opening as the loading decreased. Moreover, it is clear that the crack closed completely when *K* was 4.33 MPa·m^0.5^, as shown in Figure 10f. However, the loading was reduced to the minimum level when the crack closed completely.

### 4.2. Unstable Fatigue Crack Propagation

After the in-situ SEM testing, it can be seen that the crack began to open when the loading was larger than the crack opening stress level during the loading path, and crack tip blunting can be observed. After that, the crack gradually closed as the loading reduced during the unloading path.

**Figure 11:**
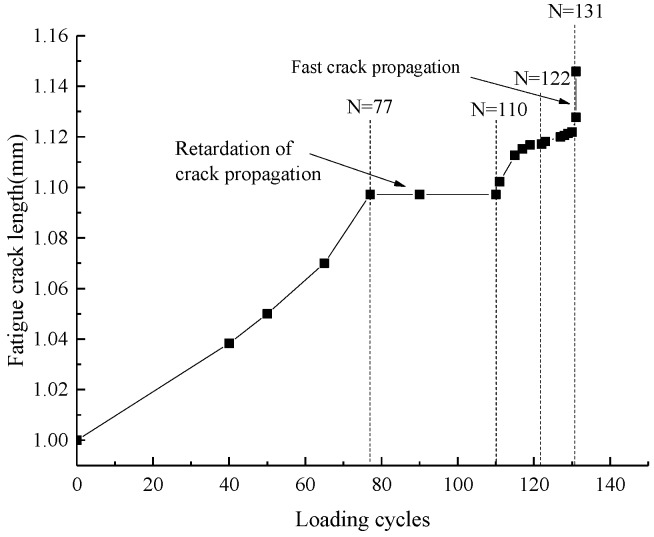
Unstable fatigue crack propagation of aluminum alloy 7050-T7451.

In this paper, the behavior of unstable fatigue crack propagation was observed across more than 100 loading cycles by in-situ SEM testing. From Figure 11, it can be seen that the crack propagation was not stable. The unstable fatigue crack growth consisted of four stages. Firstly, the crack remained at a stable growth rate until the 77th cycle. Then, the fatigue crack stopped propagating for 33 cycles. Following this, the fatigue crack restarted propagating in the 110th cycle. Finally, a fast fatigue crack growth rate was observed after the 131th cycle. However, in the macroscopic cycle-based approach, the crack propagation is stable in Paris’ region (approximately linear). However, the unstable fatigue crack propagation cannot be observed using the cycle-based approach under constant amplitude loading because of the large time scale, thus the fatigue crack growth rate increases steadily throughout the macroscopic observation. In this study, the retardation of crack propagation and fast crack propagation were closely related to the crack closure and microstructure of aluminum alloy 7050-T7451, and then the effect of the microstructure on crack growth and the relationship between CTOD and SIF during one loading cycle were investigated.

**Figure 12:**
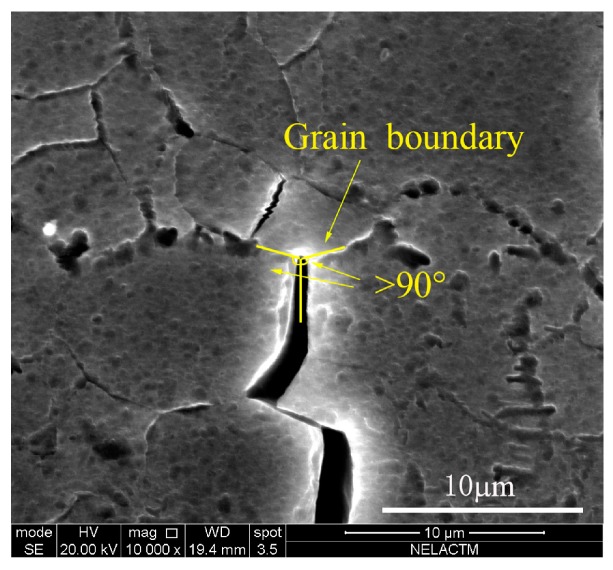
The morphology of crack tip in the 77th cycle.

As shown in Figure 12, the crack tip arrived at the grain boundary in the 77th cycle, and the angle between the grain boundary and crack was larger than 90°. After 13 loading cycles, the crack tip still remained at the grain boundary in the 90th cycle; thus, the fatigue crack propagation was blocked obviously by the grain boundary [18].

**Figure 13:**
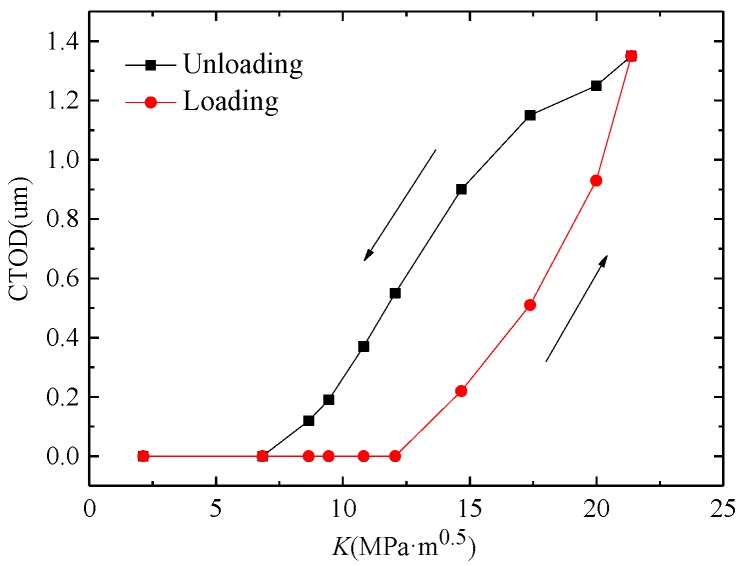
CTOD variation in the 90th cycle.

The fatigue crack propagation was analyzed by using SEM in the 90th cycle. As shown in Figure 13, the CTOD remained at zero until *K* was larger than 12.06 MPa·m^0.5^ in the 90th cycle, and the corresponding stress was 120 MPa. The CTOD increased continuously as *K* increased, and the maximum value of CTOD was 1.35 µm under maximum loading. During the unloading path, the CTOD decreased with the reduction of *K*. The crack closed completely when *K* was smaller than 6.83 MPa·m^0.5^, and the corresponding stress was 68 MPa.

**Figure 14:**
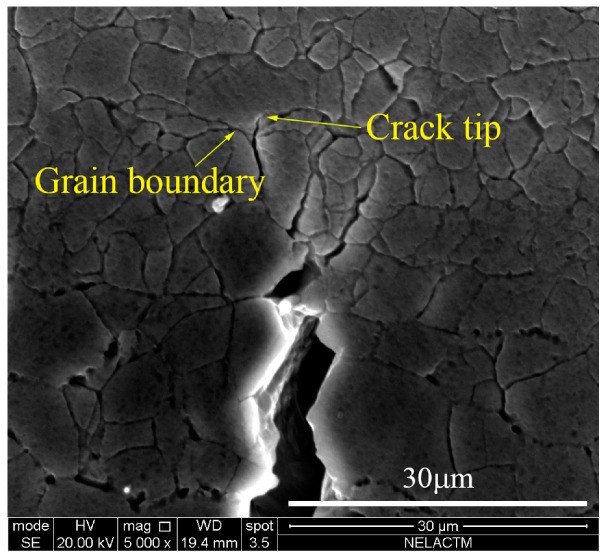
The morphology of the crack tip in the 120th cycle.

After 20 loading cycles, the fatigue crack started to propagate in the 110th cycle. The fatigue crack propagation was blocked by the grain boundary from the 77th cycle to 110th cycle. Then the crack tip propagated along the cracked grain boundary after the 110th cycle. As shown in Figure 14, the fatigue crack tip penetrated into the grain in the 120th cycle.

**Figure 15:**
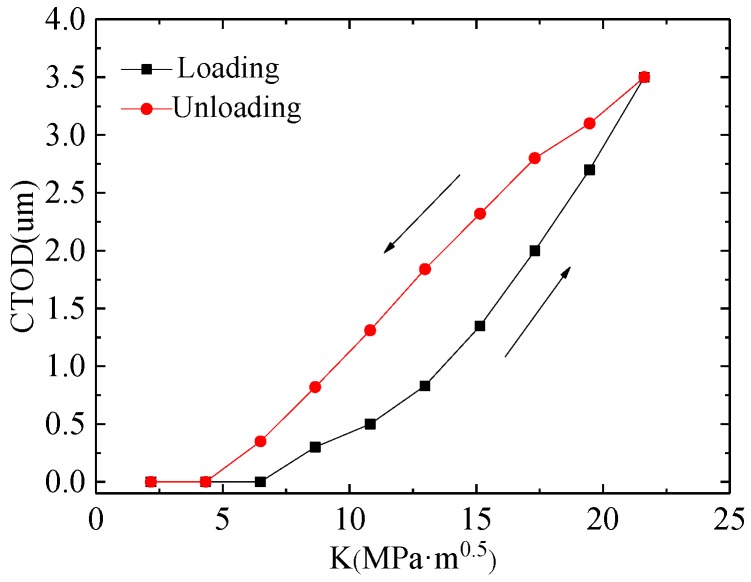
CTOD variation in the 128th cycle.

Fatigue crack propagation is closely related to the crack opening and closure behaviors, and the effective driving force of fatigue crack propagation increased as the crack opening stress level decreased [13]. From Figure 15, it can be seen that CTOD started to increase when *K* was larger than 6.49 MPa·m^0.5^, and the CTOD increased quickly as *K* increased at the loading steps in the 128th cycle. The maximum value of CTOD was 3.5 µm. Then CTOD decreased quickly, with the loading decreasing during the unloading path. Then, the crack closed completely and CTOD also reached zero when *K* was smaller than 4.33 MPa·m^0.5^.

**Figure 16:**
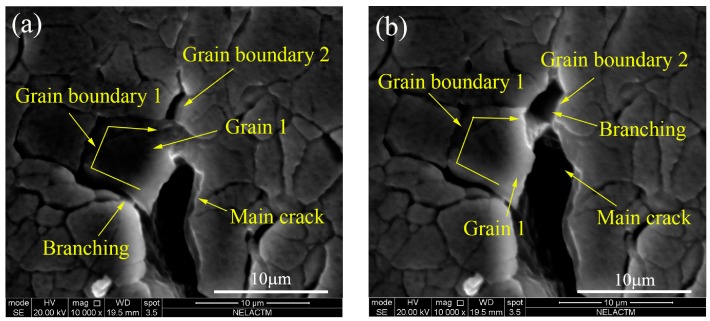
Crack branching and bridging: (**a**) crack branching in the 128th cycle; (**b**) crack bridging in the 131th cycle.

The morphology of crack propagation in the 128th cycle is shown in Figure 16a. When the crack was blocked by grain boundary 1, as shown in Figure 12, the crack was able to more easily propagate along the grain boundary than in the grain as it is known that the grain boundary is weaker than the grain. Thus, a branching crack occurred along the grain boundary of grain 1 (appears as a zigzag line in Figure 16), and the branching crack propagated quickly and arrived at grain boundary 2, which was before the main crack tip. As the main crack propagated into grain 1, the crack growth rate of the branching slowed down. In the 131th cycle, the main crack arrived at grain boundary 2 and bridged with the branching crack, as shown in Figure 16b. The bridging between the main crack tip and the branching crack resulted in the acceleration of crack propagation, and this is why the fast fatigue crack propagation of aluminum alloy 7050-T7451 occurred as shown in Figure 11.

In contrast to the 90th cycle, the CTOD of the 128th cycle started to increase when *K* was 6.49 MPa·m^0.5^, which indicates that the crack opening stress level of the 128th cycle decreased after the crack propagated into the grain. Additionally, the CTOD in the 128th cycle varied more than in the 90th cycle. Research on fatigue crack growth of aluminum alloy indicates that with the same SIF, the crack growth rate increases as CTOD increases [6]. The maximum value of CTOD in the 128th cycle was 3.5 µm, which is larger than that in the 90th cycle; hence, the crack growth rate in the 128th cycle was faster than that in the 90th cycle. This conclusion is also consistent with the results shown in Figure 11.

As discussed above, the fatigue crack growth of aluminum alloy 7050-T7451 is not stable. Before the 77th cycle, the crack propagated stably. In the 77th cycle, because the crack was blocked by the grain boundary, the crack branches and the branching crack propagated preferentially. Thus, the main crack stopped propagating transitorily, leading to retardation from the 77th cycle to the 110th cycle. Then, as the main crack propagated into the grain, the crack growth rate recovered gradually from the 110th cycle to the 130th cycle. Then, with the decrease of the branching crack growth rate and the increase of the main crack growth rate, the main crack tip bridged with the branching crack, which resulted in fast crack growth at the 131th cycle. It can be concluded that the unstable fatigue crack growth of aluminum alloy 7050-T7451 is mainly affected by the grain boundary. In aluminum alloy 7050-T7451, there are large quantities of fine, original grains and some large size, recrystallized grains. It is the fine, original grains with numerous grain boundaries that result in the unstable fatigue crack growth.

**Figure 17:**
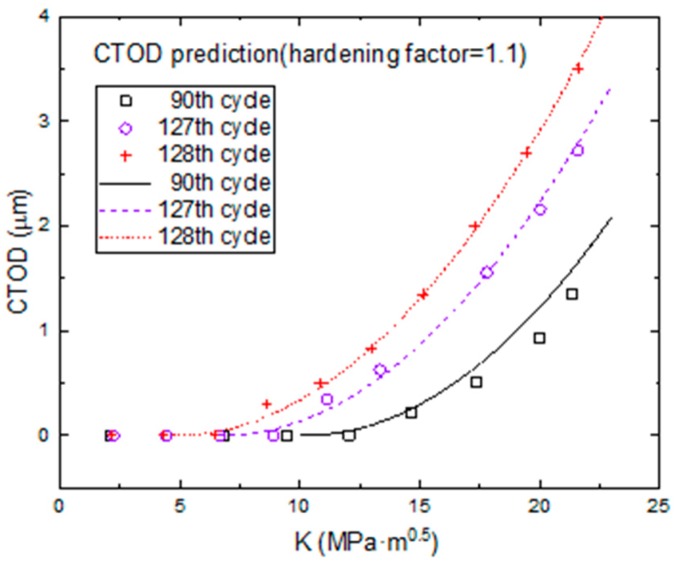
Fitted and measured CTOD variation in different loading cycles.

In the small time scale model, cracks only propagate when the applied loading exceeds the crack opening stress, and crack increments are directly correlated with CTOD variation. So, a CTOD prediction model was used to predict the CTOD variation with the *K* increase in one loading cycle. The model predictions and experimental data are shown in Figure 17. All lines represent model predictions and all points represent experimental data. In this study, there was no obvious material hardening, so the material hardening parameter (α) was set as 1.1. The effects of data randomness and averaged parameter values of Equation (4) resulted in some differences between the model predictions and experimental data. The maximum CTOD of the 90th cycle was the lowest compared with those of 127th cycle and 128th cycle under maximum stress. Moreover, the predicted CTOD in the 128th cycle was obviously larger than that in the 127th cycle; hence, the crack growth rate increased obviously when the crack expanded into the grain after one loading cycle.

In the CTOD prediction model, the crack opening stress was 22.267% of the maximal loading in the 128th cycle and 31.034% of that in the 127th cycle; these percentages are much smaller than the 46.932% of maximal loading that occurred in the 90th cycle. That is, the crack opening stress is gradually decreased from the 90th cycle to the 128th cycle, which also indicates that the grain boundary can block fatigue crack growth.

Compared with other methods, the in-situ SEM testing based on a small time scale has the following advantages. The crack closure is usually measured by the traditional compliance method using a crack displacement gauge. The opening force is calculated at the point of 2% compliance offset from a force–displacement curve, which is an indirect measurement of crack closure. This method is not accurate because many factors can affect the results. For example, the alignment of the specimen affects the measurement. Moreover, the detailed computational process is identical for different materials, which is not reliable due to the different material properties. In this paper, the crack opening stress level was directly observed and measured within one loading cycle, which is more accurate than the method used in the traditional compliance method. Additionally, most crack closure-based models consider that a crack propagates once *K* > *K*_op_ during a loading cycle, which indicates that the crack grows during both the loading and unloading paths. However, the in-situ SEM testing based on a small time scale confirmed that the crack begins to open when *K* > *K*_op_ during both the loading and unloading paths, but the crack only grows during the loading path. Therefore, the method proposed in this paper is of great significance to allow comprehensive investigation of fatigue crack growth behavior and mechanisms.

## 5. Conclusions and Future Work

In this paper, in-situ SEM testing with a small time scale was proposed to integrally analyze fatigue crack growth behavior and mechanisms. In the proposed methodology, in-situ SEM testing was used to perform small time scale loading and to investigate the effect of microstructure on fatigue crack growth. Images of the crack tip were taken to measure the CTOD and to investigate the correlation between CTOD and SIF. A CTOD model was used to predict the variation in CTOD during one loading cycle. In-situ SEM testing based on a small time scale can be used to comprehensively study the factors related to fatigue crack growth at the same time. Finally, the method was employed to investigate unstable fatigue crack growth of aluminum alloy 7050-T7451 which cannot be observed using the cycle-based approach.

In-situ SEM testing based on a small time scale is preferable to the traditional cycle-based approach. Firstly, on the basis of the experimental results, this method can be applied to quantitatively study fatigue crack growth behavior and mechanisms, such as the crack opening and closure. Secondly, the influence of the microstructure on fatigue crack growth at any time instant during one loading cycle can be observed; thus, it is more suitable for studying the crack growth behavior and mechanisms during overload. Moreover, the crack increments are directly related to CTOD variation; thus, based on the CTOD prediction model, the small time scale crack growth model can be established, which shows a continuous relationship between the fatigue crack increment (*da*) and the applied loading (*dK*/*dσ*) without considering loading cycles. Then, the crack length at any arbitrary time can be calculated through the integration of the instantaneous crack growth rate (*da*/*dt*).

## Figures and Tables

**Table 1:** Chemical composition of aluminum alloy 7050-T7451.

Element	Weight Percentage
Al	Bal.
Zn	5.9
Mg	2.29
Cu	2.11
Zr	0.13
Fe	0.051
Ti	0.026
Si	0.021
Cr	0.009
Mn	0.008

**Table 2:** Mechanical properties of aluminum alloy 7050-T7451.

Mechanical Properties	Value
Ultimate strength, *σ_b_* (MPa)	558
Yield strength, *σ_y_* (MPa)	494
Elastic modulus, *E* (GPa)	73
Fracture toughness, *K_IC_* (MPa)	38.15
Poisson’s ratio, *ν*	0.33
